# High-power short-duration vs conventional radiofrequency ablation for atrial fibrillation in patients over 80 years of age: A propensity-score matched cohort analysis

**DOI:** 10.1016/j.hroo.2025.05.018

**Published:** 2025-05-22

**Authors:** Alex Tunsch Martinez, Raphael Spittler, Nico Erhard, Florian Englert, Jan Syväri, Hannah Krafft, Miruna Popa, Theresa Reiter, Dominic Dischl, Eva Koops, Marta Telishevska, Sarah Lengauer, Gabriele Hessling, Isabel Deisenhofer, Fabian Bahlke

**Affiliations:** 1 Department of Electrophysiology, TUM University Hospital German Heart Center, Munich, Germany; 2 Center of Cardiology, Department of Cardiology II – Electrophysiology, University Medical Center Mainz, Mainz, Germany

**Keywords:** Atrial fibrillation, Ablation of Elderly, Catheter ablation, High-power short-duration ablation, Propensity score matching

## Abstract

**Background:**

Data on atrial fibrillation (AF) ablation using high-power short-duration (HPSD) ablation in patients over 80 years are lacking.

**Objective:**

This study aimed to compare the safety and efficacy of paroxysmal and persistent AF ablation using an HPSD (60–90 W/4–8 sec) vs conventional ablation (30–40 W/30 sec) in a propensity score-matched cohort of patients older than 80 years.

**Methods:**

Overall, 232 consecutive patients aged over 80 years undergoing AF ablation were included. Propensity score matching revealed 102 pairs for maximizing comparability. A post-ablation 42-day blanking period was applied. Major complications were defined as those requiring intervention or causing sequels within 30 days following catheter ablation.

**Results:**

Procedural duration (132.7 ± 45.7 vs 155.4 ± 59.7 min; *P* = .0062) and x-ray exposure (dose area product 248.9 ± 313.6 vs 544.9 ± 704.7 cGycm^2^; *P* = .0002) were significantly reduced in the HPSD group. Safety end points showed no significant differences (4/102 vs 7/102; *P =* .54). Freedom from any arrhythmia was not different between the groups including a follow-up of 22.5 ± 22.7 months (hazard ratio, 0.71; 95% confidence interval, 0.48–1.06 for the HPSD approach). After 1.52 ± 0.79 procedures, 80.4 % of all patients remained in sinus rhythm with a lower number of repeat procedures in the HPSD group (1.38 ± 0.65 vs 1.65 ± 0.90; *P* = .038).

**Conclusions:**

In very old patients (>80 years), AF ablation using an HPSD approach can be performed safely and effectively with a reduction of procedure duration, x-ray exposure, and fewer repeat ablations compared to a conventional approach. Long-term ablation results are promising with both approaches.

## Graphical abstract

**Figure:**
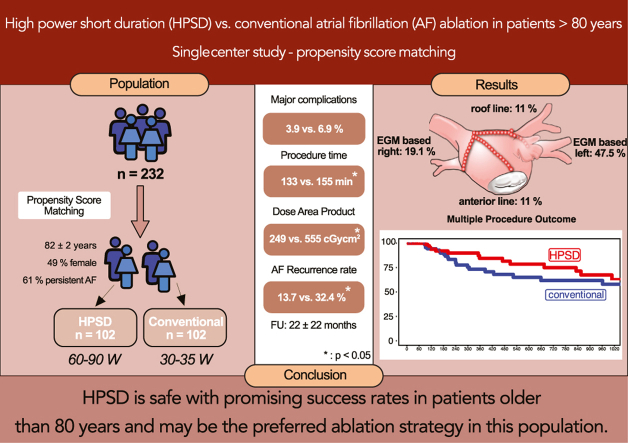

**Key Findings**

▪ High power short duration (HPSD) ablation reduces the procedural burden in older patients over 80 years by decreasing procedure duration, x-ray exposure, and the need for re-ablation.
▪ HPSD ablation maintains a low complication rate in this frail patient cohort (3.9%).
▪ Long-term success rates (80.4% after 1.52 ± 0.79 procedures) are promising in patients >80 years.

## Introduction

Atrial fibrillation (AF) is the most prevalent arrhythmia in industrialized countries, with increasing incidence in aging societies.^1^^,^^2^ This demographic shift is expected to lead to a growing number of older patients requiring rhythm control strategies.^1^

Although success rates following catheter ablation are often reported to be lower in an elderly population,^3^, ^4^, ^5^ some studies suggest comparable outcomes to those in younger patients.^6^ These contradictory observations may be influenced by variations in ablation techniques, approaches, and patient characteristics. Achieving optimal long-term success in elderly patients is often constrained by age-associated atrial remodeling, which might limit the efficacy of interventional rhythm control strategies.^7^ In large studies, very old patients (> 80 years) are frequently underrepresented.^8^^,^^9^ and there is also a lack of data on new ablation approaches such as high-power short-duration (HPSD) ablation for this age cohort. HPSD ablation is considered to reduce procedure duration and improve lesion durability through reduced conductive and enhanced resistive heating^10^ and may result in higher first-pass isolation rates.^11^ HPSD lesions are characterized by larger diameter and shallower depth, which may reduce the risk of collateral damage to structures such as the esophagus or other tissues surrounding the left atrium.^10^^,^^12^, ^13^, ^14^

As there are no published data on the use of HPSD ablation in patients over 80 years, this study aimed to provide clinical data on the safety and efficacy of HPSD compared to a conventional ablation approach.

## Methods

### Study population

In this single-center study, 232 consecutive patients aged over 80 years undergoing their first ablation for paroxysmal (n = 86) or persistent (n = 146) AF at the German Heart Center Munich between 2016 and 2021 were included. Patients receiving repeat procedures were excluded. Paroxysmal and persistent AF were classified according to current guidelines.^15^^,^^16^ Based on ablation parameters, 2 groups of patients were defined. The first group underwent HPSD ablation with 60–90 Watt (W) for 4–8 seconds while the second group underwent a conventional ablation approach using 30–40 W applied for 20–40 seconds per lesion. Ethical approval for the study was obtained from the local ethics committee, and the study was conducted in accordance with the Declaration of Helsinki on human research. Patients provided written informed consent for the ablation procedure and data processing for study purposes.

### Propensity score matching

To minimize confounding bias and ensure balanced baseline characteristics between the groups, propensity score matching was performed with the following matching variables: AF type, gender, body mass index (BMI), left ventricular ejection fraction, and cardiovascular risk factors (hypertension, diabetes, and stroke). The propensity score was estimated using logistic regression and the matching was conducted using optimal matching with a caliper width of 0.2 standard deviations of the logit-transformed propensity score to restrict matches to those with similar propensity scores, reducing residual bias. Patients were matched in a 1:1 ratio without replacement. To evaluate the effectiveness of the matching process, several diagnostic checks were performed: standardized mean differences (SMDs): Computed for each covariate before and after matching, with an SMD and an empirical cumulative distribution function mean differences <0.1 considered indicative of adequate balance. Visual control for optimal matching was performed by calculation of several plots (QQ-, jitter, and propensity score histograms).

After propensity score matching, a total of 102 pairs were identified. Due to the matching process, observed baseline covariates are comparable between the 2 groups.

### Procedural workflow

A standardized workflow for atrial ablation procedures was conducted for all patients. Oral anticoagulation was maintained uninterrupted before ablation. Anti-arrhythmic drugs were discontinued at least 5 half-lives before the procedure. All patients underwent transesophageal echocardiography or cardiac computed tomography to exclude atrial thrombus formation less than 48 hours before the intervention. Procedures were performed under deep sedation, using propofol, fentanyl and/or midazolam with a non-invasive blood pressure monitoring. Double left atrial access was gained after triple punction of the femoral vein and single transseptal puncture. The single transseptal puncture was conducted with a steerable sheath (Agilis, Abbott, Minneapolis, MN, USA). Following double left atrial access heparin was administered in a weight-adapted dose. Activated clotting time was measured at least twice an hour, aiming levels of an activated clotting time>330 seconds.

In all cases, the left atrium was mapped with a multipolar catheter using an electroanatomical 3D-mapping system (either NavX Precision/Ensite X, Abbott, Minneapolis, USA or Carto3, Biosense Webster, Irvine, USA). Wide area circumferential ablation pulmonary vein isolation (PVI) was performed in all cases. In the presence of atrial tachycardia (AT), entrainment maneuvers and/or a local activation time mapping were used to reveal the underlying AT mechanism, followed by linear lesions or focal ablation. Only a point-by-point ablation approach using radiofrequency energy was used. HPSD ablation was defined as point-by-point ablation using 60–90 W depending on the used ablation catheter for 4–8 seconds. In the conventional ablation group, 30–40 W were applied for 30–40 seconds on the anterior wall of the left atrium and for 15–20 seconds on posterior left atrial sites. The target baseline impedance in the left atrium before ablation was 120 Ohm in all patients.

Procedural end point for successful PVI was proof of entry- and/or exit-block of all pulmonary veins. Adenosine was used to identify dormant PV conduction, in the absence of contraindications. Persistent bidirectional block of created linear lesions was evaluated by differential pacing. To test further AT inducibility, burst stimulation was performed after AT termination. A purse-string suture was used to stop bleeding after removing the venous sheaths.

### In-hospital monitoring after catheter ablation

On the day following ablation, pericardial effusion was excluded. Duplex sonography of the groin vessels was performed in all patients whenever available, with additional examinations in cases of suspected puncture site complications. Any in-hospital neurological deficit after a procedure was examined by a neurologist. Transient ischemic attack (TIA) was defined as acute focal neurological symptoms without correlation in imaging and less than 24-hour duration of symptoms. Stroke was defined as acute neurological deficit with evidence of ischemia or hemorrhage on brain imaging. For prevention of esophageal injury after left atrial ablation, all patients received proton pump inhibitors (typically pantoprazole 40 mg twice daily) for 4 weeks.

### Follow-up

Follow-up visits were scheduled 1, 3, and 12 months after ablation, including repetitive 7-day Holter-electrocardiography. Telephone interviews were conducted if patients missed appointments. Any atrial arrhythmia lasting longer than 30 seconds was classified as recurrence, after a blanking period of 42 days. Safety analysis included complications requiring intervention or causing sequelae within 30 days of the procedure.

### Primary efficacy and safety end points

The primary efficacy end point consisted of a documented episode of any atrial arrhythmia lasting longer than 30 seconds and occurring after a 42-day blanking period following the initial procedure. Safety end points were defined as periprocedural death, stroke, TIA, pericardial tamponade, major groin complication with the need for intervention, and vascular embolism.

### Statistical analysis

Continuous variables were expressed as mean ± standard deviation if normally distributed otherwise as median with interquartile range and analyzed using the Student *t* test or Mann-Whitney U test. Categorical variables were presented as absolute and relative frequencies (percentage) and analyzed with the χ2 or Fisher exact test. Kaplan-Meier survival analysis was conducted to assess arrhythmia probability over time, that is, recurrence rates within the follow-up period. Differences were evaluated using the log-rank test. A *P* value of < .05 was considered statistically significant. A logistic regression analysis was performed with treatment and operator as independent variables to analyze the possible effect of different operators on the end points variables. The statistical analysis was performed using SPSS Statistics software (SPSS Statistics 29, IBM, USA) and R (Version 12.1, R Core Team, Austria).

## Results

### Baseline characteristics

After propensity score matching, 102 patients undergoing HPSD ablation were matched with 102 patients who underwent conventional ablation, yielding 2 well-balanced cohorts.

Baseline characteristics of the 204 patients including age (82.2 ± 2.1 years), sex distribution (49% women), the prevalence of comorbidities such as hypertension (HPSD: 90.2 % vs conventional 92.2 %, *P* = .81), diabetes (18.6 % vs 18.6 %, *P =* 1) or BMI (26.0 ± 4.7 vs 26.2 ± 4.4, *P =* .4) were comparable between groups, with no significant differences observed. The mean CHA_2_DS_2_-VA-score (3.9 ± 1.1 vs 4.1 ± 1.2, *P =* .39), and mean LA size (28.5 ± 9.2 vs 27.9 ± 7.2 mm, *P =* .87) were comparable as well. The baseline characteristics of both matched groups are listed in Table 1.

**Table 1:** Baseline characteristics

	Overall population (n = 204)	HPSD approach (n = 102)	Conventional approach (n = 102)	*P* value	OR (95% CI)
Age (y)	82.2 ± 2.1	82.2 ± 2.1	82.2 ± 2.0	.84	—
Women, n (%)	100 (49)	50 (49)	50 (49)	1	1.0 (0.6–1.8)
BMI, (kg/m^2^)	26.1 ± 4.6	26.0 ± 4.7	26.2 ± 4.4	.41	—
Persistent AF, n (%)	124 (61)	63 (62)	61 (60)	.89	0.9 (0.5–1.7)
CHA_2_DS_2_-VA-score	4.0 ± 1.1	3.9 ± 1.1	4.1 ± 1.2	.39	—
Previous stroke or TIA, n (%)	31 (14.7)	12 (11.8)	19 (18.6)	.24	1.7 (0.7–4.1)
Diabetes, n (%)	38 (18.6)	19 (18.6)	19 (18.6)	1	1.0 (0.5–2.2)
Hypertension, n (%)	186 (91.2)	92 (90.2)	94 (92.2)	.81	1.3 (0.4–3.9)
Heart failure, n (%)	50 (24.5)	30 (29.4)	20 (19.6)	.14	0.6 (0.3–1.2)
LVEF (%)	52.7 ± 8.8	52.5 ± 8.4	52.9 ± 9.3	.38	—
LA size (cm^2^)	28.1 ± 8.1	28.5 ± 9.2	27.9 ± 7.2	.87	—
eGFR (mL/min)	61.2 ± 17.5	61.3 ± 17.0	62.0 ± 18.5	.79	—

Values are given as n (%), mean ± SD or median (min–max).

BMI = body mass index; eGFR = estimated glomerular filtration rate; HPSD = high-power short-duration; LA = left atrium; LVEF = left ventricular ejection fraction; OR = odds ratio; TIA = transient ischemic attack.

### Procedural data

Total procedure time (132.74 ± 45.64 vs 155.4 ± 59.7 min, *P =* .006) was significantly lower in the HPSD group. Fluoroscopy time was insignificantly longer in the conventional group (8.5 ± 5.4 vs 9.9 ± 6.8 min, *P =* .16) but x-ray exposure (dose area product HPSD: 248.9 ± 313.6 vs 554.9 ± 708.5 cGycm,^2^ *P <* .0001) was significantly lower in the HPSD group. As expected, radiofrequency time was significantly lower in the HPSD group (24.2 ± 11.5 min vs 48.7 ± 22.9 min, *P <* .0001) whereas radiofrequency (RF) power was significantly higher in the HPSD group (57.3 ± 7.7 W vs 32.0 ± 2.6 W, *P <* .0001). Procedural data are shown in Table 2.

**Table 2:** Procedural data

	Overall population (n = 204)	HPSD approach (n = 102)	Conventional approach (n = 102)	*P* value
Procedure duration (min)	144.1 ± 54.2	132.7 ± 45.7	155.4 ± 59.7	.0062
Radiofrequency duration (min)	36.4 ± 21.9	24.2 ± 11.5	48.7 ± 22.9	<.0001
Fluoroscopy time (min)	9.2 ± 6.2	8.5 ± 5.4	9.9 ± 6.8	.16
DAP (cgycm^2^)	401.9 ± 567.6	248.9 ± 313.6	544.9 ± 704.7	.0002
Ablation power (Watt)	44.8 ± 14.0	57.3 ± 7.7	32.0 ± 2.6	<.0001
Tip temperature (Celsius)	30.5 ± 4.8	30.3 ± 4.9	30.7 ± 4.8	.82

Values are given as mean ± SD.

DAP *=* dose area product; HPSD = high-power short-duration.

The procedural end point of PVI was achieved in all patients. Additional ablation such as left atrial lines, electrogram-based substrate modification, or cavotricuspid isthmus ablation were equally distributed between the 2 groups (Table 3).

**Table 3:** Ablation lesions

	Overall population (n = 204)	HPSD approach (n = 102)	Conventional approach (n = 102)	*P* value	OR (95% CI)
Pulmonary vein isolation, n (%)	204 (100)	102 (100)	102 (100)	1	—
Electrogram-based substrate modification in left atrium, n (%)	97 (47.5)	53 (52.0)	44 (43.1)	.26	0.7 (0.4–1.3)
Electrogram-based substrate modification in right atrium, n (%)	39 (19.1)	16 (15.7)	23 (22.5)	.29	1.6 (0.7–3.4)
Electrogram-based substrate modification in coronary sinus, n (%)	41 (20.1)	20 (19.6)	21 (20.6)	1	1.1 (0.5–2.2)
Anterior line, n (%)	23 (11.3)	11 (10.8)	12 (11.8)	1	1.1 (0.4–2.9)
Roof line, n (%)	23 (11.3)	10 (9.8)	13 (12.7)	.66	1.3 (0.5–3.6)
Ablation of cavotricuspid isthmus, n (%)	43 (21.1)	18 (17.6)	25 (24.5)	.30	1.5 (0.7–3.2)

Values are given as n (%).

HPSD = high-power short-duration; OR = odds ratio.

### Safety

Major complications occurred in 5.3% (11/204) of patients in the total cohort, with similar rates in the HPSD and conventional group (Table 4). The most frequent adverse events were vascular access complications (4.4%). One TIA occurred in the HPSD group and 1 case of cardiac tamponade requiring pericardiocentesis in the conventional ablation group.

**Table 4:** Safety end points

	Overall population (n = 204)	HPSD approach (n = 102)	Conventional approach (n = 102)	*P* value	OR (95% CI)
Major complication, n (%)	11 (5.3%)	4 (3.9%)	7 (6.9%)	.54	1.8 (0.4–8.7)
Pericardial tamponade, n (%)	1 (0.5%)	0	1 (1.0%)	—	—
Groin complication, n (%)	9 (4.4%)	3 (2.9%)	6 (5.9%)	.49	2.1 (0.4–13.1)
Transient ischemic attack, n (%)	1 (0.5%)	1	0	—	—
Stroke, n (%)	0	0	0	—	—
Death, n (%)	0	0	0	—	—

Values are given as n (%).

HPSD = high-power short-duration.

Minor complications including hematomas (5/102 in HPSD group vs 2/102 in conventional group, *P =* .44) and small pericardial effusion not requiring an intervention (3/102 vs 2/102, *P =* 1), were similar between the groups.

### Efficacy

**Figure 1:**
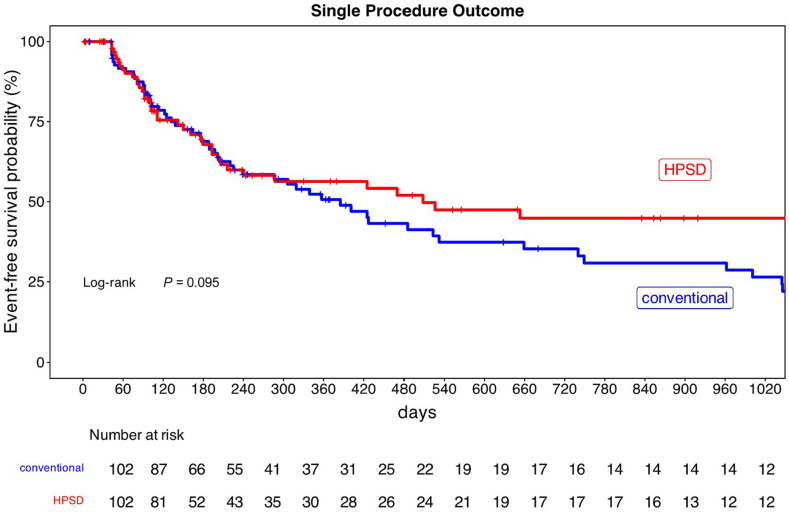
Kaplan-Meier curve of arrhythmia-free survival in the high power short duration (HPSD) group and in the conventional group after one ablation procedure. Any atrial tachycardia during follow-up was defined as recurrence. Log-rank test revealed no significant difference in both groups (*P* = .095).

After a mean follow-up of 22.5 ± 22.7 months, atrial arrhythmia recurrence occurred in 36 patients (35.2 %) in the HPSD group compared to 57 patients (55.8 %) in the conventional ablation group (odds ratio [OR], 0.43; 95% confidence interval [CI], 0.24–0.79; *P =* .0048). After 12 months, 64.7 % of all patients were in stable sinus rhythm. Kaplan-Meier survival analysis demonstrated a higher arrhythmia-free survival rate after 1 ablation in the HPSD cohort without reaching statistical significance (log-rank *P* = .095; hazard ratio [HR], 0.71 [95% CI, 0.48–1.06], Figure 1). Type of arrhythmia recurrence differed between the groups: AF recurrences occurred significantly less often in the HPSD group (13.7% vs 32.4%; *P =* .0025), whereas AT recurrences were comparable (21.5% [HPSD] vs 23.5% [conventional]; *P =* .87).

**Figure 2:**
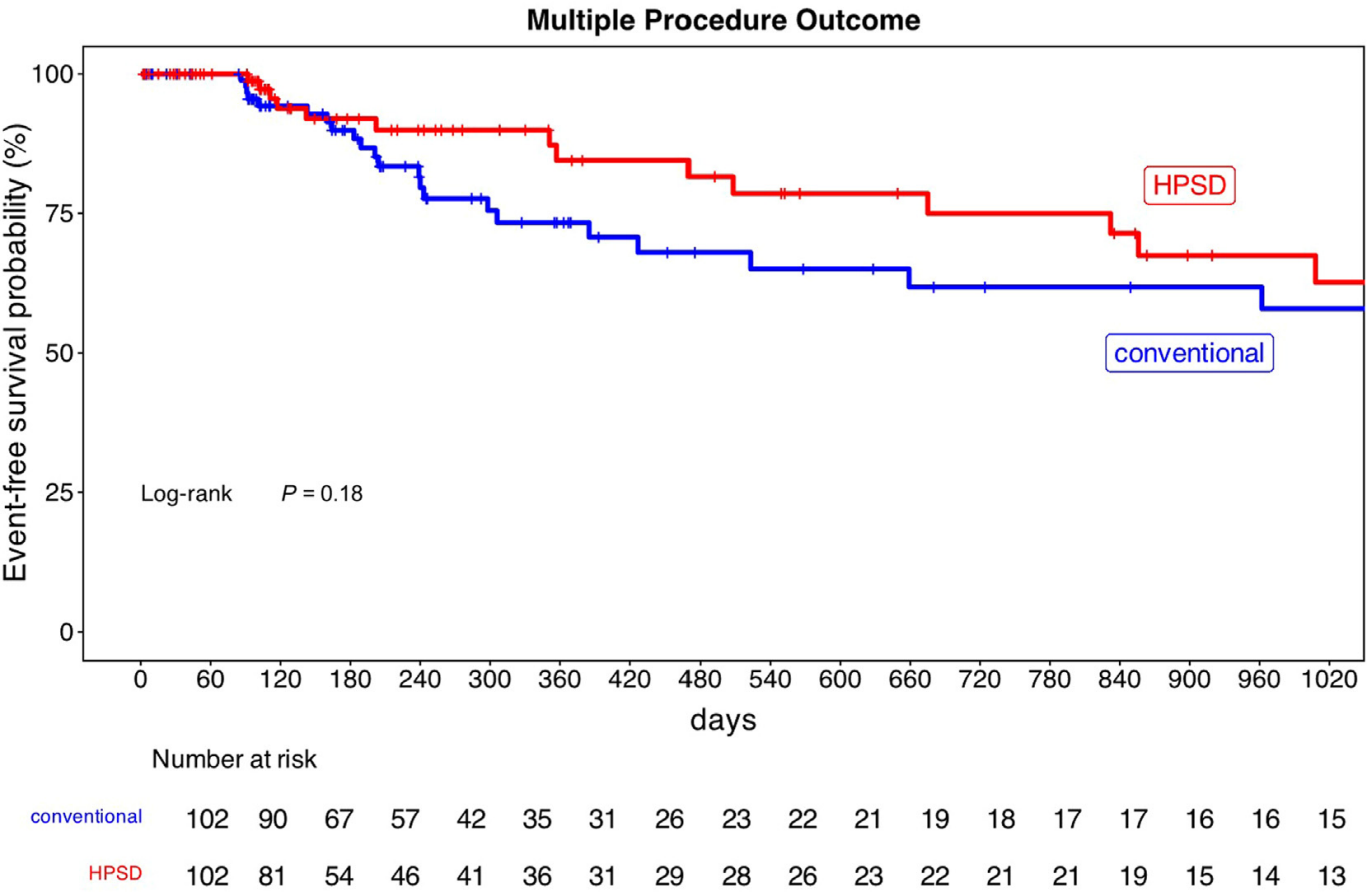
Kaplan-Meier curve of arrhythmia-free survival in the high power short duration (HPSD) group and in the conventional group after multiple ablation procedures (mean number of procedures 1.52 ± 0.79). Any atrial tachycardia after the last procedure was defined as recurrence. Log-rank test revealed no significant difference in both groups (*P* = .18).

After 1.52 ± 0.79 procedures, 80.4 % of patients remained in sinus rhythm. The number of repeat procedures was lower in the HPSD group (1.38 ± 0.65 vs 1.65 ± 0.90; *P =* .038). Recurrences of any atrial arrhythmia after the last procedure were not significantly different in both groups (14/102 vs 26/102, OR, 2.14 [95% CI, 0.99–4.78], *P =* .051). Outcome was comparable between HPSD and a conventional approach after multiple procedures (HR, 0.63; 95% CI, 0.32–1.24; log-rank test *P =* .18, Figure 2). During follow-up, 9 patients (4.4%) received class III antiarrhythmic drugs without differences in both groups (7 patients in HPSD group, 2 patients in conventional group; OR, 3.72, 95% CI, 0.76–18.36; *P =* .11). Additionally, the Fisher exact test revealed no statistical differences in the use of antiarrhythmic drugs in both groups (class I–IV; *P =* .84 before and *P =* .27 after ablation)

Multivariate logistic regression analysis with the treatment and operator as independent variables revealed that only the treatment (HPSD vs conventional ablation) had a significant effect on dose area product, procedure time, and outcome but not the operator (effect on outcome: *P =* .031 for treatment; *P =* .32 for the operator).

## Discussion

To the best of our knowledge, this study provides the first analysis of the safety and efficacy of HPSD ablation in comparison to conventional RF ablation in very old patients (>80 years) using propensity score matching. HPSD reduces the procedural burden (procedure time, radiation exposure) in this patient cohort while maintaining low complication rates. A high long-term success rate of 80.4% was reached with both approaches after 1.52 ± 0.79 procedures which is a very promising result for patients >80 years with AF.

### Procedural data and safety

HPSD ablation significantly reduces procedure time and x-ray exposure compared to conventional ablation, which is particularly relevant in older patients to minimize procedural burden. Recently published meta-analyses also revealed shorter procedure times and shorter RF duration in HPSD ablation.^13^^,^^17^ Notably, procedure duration in this study was comparable to published multi-center trials and meta-analyses,^8^^,^^13^^,^^17^^,^^18^ implicating that catheter ablation of old and frail patients can be performed without prolonging procedure duration. Shorter procedure times lead to a reduction of sedation or anesthesia and time in the left atrium. Thus, the risk of periprocedural stroke as a relevant cause of death following AF ablation is reduced.^13^^,^^19^^,^^20^ A lower procedural burden seems crucial as this age cohort tends to have higher complication rates following AF ablation.^21^ The major complication rate was low (11/204, 5.4%) and comparable between HPSD ablation and conventional ablation.

Femoral vessel complications were reported slightly lower between 0.8%–3.1%,^9^^,^^22^, ^23^, ^24^, ^25^, ^26^ which might be explained by lower diameters of femoral veins with increasing age^27^ and uninterrupted oral anticoagulation in all patients. Routine sonography of the groin on the day following ablation might lead to a surveillance bias. Given the increased risk of femoral vessel complications, sonography-guided puncture seems essential.^24^

One TIA occurred in the HPSD group and one pericardial tamponade had to be treated in the conventional group. No other major complications, such as stroke, phrenic nerve palsy, atrio-esophageal fistula, or periprocedural death occurred. Thromboembolic events were rare, highlighting the benefits of uninterrupted oral anticoagulation.^28^^,^^29^ This is particularly relevant as 15% of the cohort had a history of stroke and were considered a high-risk population.

### Efficacy of HPSD ablation

Overall recurrence rates were lower in the HPSD group compared to the conventional group (35.2% vs 55.8%; *P =* .0048). However, the log-rank test showed no difference in recurrence-free survival when assessing recurrence probability over time using Kaplan-Meier analysis during 686.7 ± 674.5 days of follow-up (*P =* .1). Interestingly, the overall reduction of recurrences was driven by a significantly lower rate of AF recurrence in the HPSD group (13.7% vs 32.4%; *P =* .0025), while AT recurrence was similar in both groups (21.5% vs 23.5%; *P =* .87).

These findings suggest that HPSD ablation may lead to more durable and sufficient PVI as a result of more consistent lesion formation and reduced procedural variability.^30^ A recent study by Liu et al.^31^ showed higher rates of first-pass isolation using HPSD ablation, reducing the likelihood of pulmonary vein reconnections as key drivers of AF recurrence.^32^, ^33^, ^34^ This is consistent with a meta-analysis of randomized controlled trials comparing HPSD ablation with conventional RF ablation.^13^ Notably, fewer repeat procedures were necessary to achieve a similar long-term success using HPSD ablation (Figure 2), highlighting a further reduction in procedural burden.

The comparable AT recurrence rates suggest that HPSD ablation does not provide additional benefit following electrogram-based substrate modification in persistent AF, likely due to ablation-related fibrosis and progressive atrial remodeling.^34^^,^^35^ Since the ablation protocol itself was identical in both groups, the alteration of the individual atrial substrate may have a greater influence on AT recurrence rather than the ablation technique.

### Long-term efficacy

The detailed analysis of long-term follow-up in the overall patient cohort yielded 2 key findings. First, 93/204 patients (45.5%) experienced a recurrence after a single procedure during a follow-up time of 672.6 ± 682.69 days, with 35% of patients experiencing a recurrence after 12 months. Comparing these results with previously published studies is difficult because of several factors including differences in baseline characteristics, the proportion of patients with persistent AF, and differences in the quality and duration of follow-up. However, our single-procedure success rates seem comparable to earlier studies. Boehmer et al.^4^ observed 33.2% recurrences after 12 months in patients aged over 75 years (39% persistent AF), Bulava et al.^36^ reported 36% recurrences in patients >80 years of age (58% persistent AF) and Vermeersch et al.^37^ described 63.9% recurrences after median follow-up of 24 months in patients aged over 75 years (all patients with persistent AF). Müller et al.^38^ compared safety and efficacy of HPSD in the elderly (> 75 years) and younger patients and reported a recurrence rate of 34% after 1 year (90 patients, 40% persistent AF).

Given our comprehensive follow-up protocol, which consisted of repeated Holter-electrocardiography, a mean follow-up of approximately 2 years, and a substantial proportion of persistent AF (61%) patients, success rates after a single procedure seem promising. Moreover, cumulative success rates of 80.4% after 1.51 ± 0.79 ablations seem very favorable. Based on these outcomes, interventional rhythm control should be considered in all patients over 80 years as an alternative to amiodarone treatment which has a high risk of adverse effects, especially in older patients.^39^^,^^40^ The success rates in our cohort were somewhat unexpected and promising considering pathophysiological concepts on atrial cardiomyopathy and that “AF begets AF”.^41^^,^^42^ Age is recognized as a risk factor for arrhythmia recurrence following catheter ablation.^43^ Selection bias might play a role as a mean CHA_2_DS_2_-VA-Score of 4.0 ± 1.1 may indicate a patient cohort lacking a variety of comorbidities, given that age >75 years contributes 2 points to the total score. In contrast, more than half of our population were women, which has been reported as a risk factor for arrhythmia recurrence following AF ablation.^43^ Interestingly, other trials also reported a higher ratio of women in elderly patient cohorts undergoing AF ablation, whereas women are usually underrepresented in younger patient cohorts.^5^^,^^8^^,^^44^, ^45^, ^46^

Further large trials and comparisons of new approaches including pulsed-field ablation or a pace-and-ablate strategy are needed to evaluate the best treatment options for older patients.^47^

## Limitations

This is a single-center study with all inherent limitations. Although propensity score matching was used to enhance comparability between groups, it does not fully substitute a randomized control trial. Entry block confirming PVI was demonstrated in all patients following the recent expert consensus^15^^,^^48^; however, the number of exit-block was not documented which is a potential bias.

We cannot exclude that different operators might have influenced the results, but as 2 physicians were supervising all procedures and operators did not differ significantly between the groups, we assume that the probability of the bias influencing the results is low.

Only clinically relevant complications were evaluated. The study is underpowered for rare complications such as esophageal fistula or periprocedural death.

## Conclusions

Catheter ablation of AF can be performed safely and effectively in patients over the age of 80 years. HPSD ablation showed promising data, especially regarding the reduction of procedural burden and might be the superior approach in this age group. Old age does not seem to limit long-term success using HPSD or conventional ablation approaches. The findings are highly relevant to clinical practice in light of an aging population with a growing number of older patients requiring rhythm control strategies.

## References

[1] Krijthe B.P., Kunst A., Benjamin E.J. (2013). Projections on the number of individuals with atrial fibrillation in the European Union, from 2000 to 2060. Eur Heart J.

[2] Kornej J., Börschel C.S., Benjamin E.J., Schnabel R.B. (2020). Epidemiology of atrial fibrillation in the 21st century: novel methods and new insights. Circ Res.

[3] Kautzner J., Peichl P., Sramko M., Cihak R., Aldhoon B., Wichterle D. (2017). Catheter ablation of atrial fibrillation in elderly population. J Geriatr Cardiol.

[4] Boehmer A.A., Rothe M., Keim C. (2024). Pulmonary vein isolation in elderly patients ≥ 75 years: a propensity score-matched analysis with focus on differences among atrial fibrillation types. Can J Cardiol.

[5] Bahnson T.D., Giczewska A., Mark D.B. (2022). Association between age and outcomes of catheter ablation versus medical therapy for atrial fibrillation: results from the CABANA trial. Circulation.

[6] Inoue K., Nakai M., Yamane T. (2025). Assessment of the safety and efficacy of catheter ablation for atrial fibrillation in very elderly patients: insight from the national prospective registry study. Eur Heart J Qual Care Clin Outcomes.

[7] Lin Y.-K., Chen Y.-A., Lee T.-I., Chen Y.-C., Chen S.-A., Chen Y.-J. (2018). Aging modulates the substrate and triggers remodeling in atrial fibrillation. Circ J.

[8] Kistler P.M., Chieng D., Sugumar H. (2023). Effect of catheter ablation using pulmonary vein isolation with vs without posterior left atrial wall isolation on atrial arrhythmia recurrence in patients with persistent atrial fibrillation: the CAPLA randomized clinical trial. JAMA.

[9] Huo Y., Gaspar T., Schönbauer R. (2022). Low-voltage myocardium-guided ablation trial of persistent atrial fibrillation. NEJM Evid.

[10] Leshem E., Zilberman I., Tschabrunn C.M. (2018). High-power and short-duration ablation for pulmonary vein isolation: biophysical characterization. JACC Clin Electrophysiol.

[11] Salló Z., Perge P., Balogi B. (2022). Impact of high-power and very high-power short-duration radiofrequency ablation on procedure characteristics and first-pass isolation during pulmonary vein isolation. Front Cardiovasc Med.

[12] Mueller J., Halbfass P., Sonne K. (2022). Safety aspects of very high power very short duration atrial fibrillation ablation using a modified radiofrequency RF-generator: single-center experience. J Cardiovasc Electrophysiol.

[13] Amin A.M., Ghaly R., Ibrahim A.A. (2024). Efficacy and safety of high-power short-duration ablation for atrial fibrillation: a systematic review and meta-analysis of randomized controlled trials. J Interv Card Electrophysiol.

[14] Ali-Ahmed F., Goyal V., Patel M., Orelaru F., Haines D.E., Wong W.S. (2019). High-power, low-flow, short-ablation duration-the key to avoid collateral injury?. J Interv Card Electrophysiol.

[15] Tzeis S., Gerstenfeld E.P., Kalman J. (2024). 2024 European Heart Rhythm Association/Heart Rhythm Society/Asia Pacific Heart Rhythm Society/Latin American Heart Rhythm Society expert consensus statement on catheter and surgical ablation of atrial fibrillation. Europace.

[16] Van Gelder I.C., Rienstra M., Bunting K.V. (2024). ESC Guidelines for the management of atrial fibrillation developed in collaboration with the European Association for Cardio-Thoracic Surgery (EACTS). Eur Heart J.

[17] Ravi V., Poudyal A., Abid Q.-U.-A. (2021). High-power short duration vs. conventional radiofrequency ablation of atrial fibrillation: a systematic review and meta-analysis. Europace.

[18] Castrejón-Castrejón S., Martínez Cossiani M., Basterra Sola N. (2025). High-power short-duration radiofrequency application for faster and safer pulmonary vein isolation: the Power-FAST III trial. JACC Clin Electrophysiol.

[19] Cappato R., Calkins H., Chen S.A. (2009). Prevalence and causes of fatal outcome in catheter ablation of atrial fibrillation. J Am Coll Cardiol.

[20] Kamioka M., Watanabe T., Watanabe H. (2024). High-power short-duration setting prevents changes of periprocedural thrombotic markers and the onset of silent stroke in patients with atrial fibrillation. Heart Rhythm O2.

[21] Mark J.D., Colombo R.A., Alfonso C.E. (2024). The impact of frailty on patients with AF and HFrEF undergoing catheter ablation: a nationwide population study. JACC Adv.

[22] Bode K., Ueberham L., Gawlik S., Hindricks G., Bollmann A. (2019). Inguinal vascular complications after ablation of atrial fibrillation: an economic impact assessment. Europace.

[23] Dagres N., Hindricks G., Kottkamp H. (2009). Complications of atrial fibrillation ablation in a high-volume center in 1,000 procedures: still cause for concern?. J Cardiovasc Electrophysiol.

[24] Foerschner L., Erhard N., Dorfmeister S. (2022). Ultrasound-guided access reduces vascular complications in patients undergoing catheter ablation for cardiac arrhythmias. J Clin Med.

[25] Deshmukh A., Patel N.J., Pant S. (2013). In-hospital complications associated with catheter ablation of atrial fibrillation in the United States between 2000 and 2010: analysis of 93 801. Circulation.

[26] Fink T., Metzner A., Willems S. (2019). Procedural success, safety and patients satisfaction after second ablation of atrial fibrillation in the elderly: results from the German ablation Registry. Clin Res Cardiol.

[27] Fronek A., Criqui M.H., Denenberg J., Langer R.D. (2001). Common femoral vein dimensions and hemodynamics including valsalva response as a function of sex, age, and ethnicity in a population study. J Vasc Surg.

[28] Kottmaier M., Bourier F., Semmler V. (2017). Catheter ablation of left atrial arrhythmias on uninterrupted oral anticoagulation with vitamin K antagonists: what is the relationship between international normalized ratio, activated clotting time, and procedure-related complications?. J Cardiovasc Electrophysiol.

[29] Cardoso R., Willems S., Gerstenfeld E.P. (2019). Uninterrupted anticoagulation with non-vitamin K antagonist oral anticoagulants in atrial fibrillation catheter ablation: lessons learned from randomized trials. Clin Cardiol.

[30] Li M.F., Wu J., Jin C.L., Chen C.F., Xu Y.Z. (2021). Safety and efficacy of high power shorter duration ablation for atrial fibrillation: a systematic review and meta-analysis. Int J Clin Pract.

[31] Liu X., Gui C., Wen W., He Y., Dai W., Zhong G. (2021). Safety and efficacy of high power shorter duration ablation guided by ablation index or lesion size index in atrial fibrillation ablation: a systematic review and meta-analysis. J Interv Cardiol.

[32] Nery P.B., Belliveau D., Nair G.M. (2016). Relationship between pulmonary vein reconnection and atrial fibrillation recurrence: a systematic review and meta-analysis. JACC Clin Electrophysiol.

[33] Das M., Wynn G.J., Saeed Y. (2017). Pulmonary vein re-isolation as a routine strategy regardless of symptoms: the PRESSURE randomized controlled trial. JACC Clin Electrophysiol.

[34] Spittler R., Bahlke F., Hoffmann B.A. (2022). Durable pulmonary vein isolation but not complex substrate ablation determines the type of arrhythmia recurrence after persistent atrial fibrillation ablation. J Interv Card Electrophysiol.

[35] Hung Y., Chang S.L., Lin W.S., Lin W.Y., Chen S.A. (2020). Atrial tachycardias after atrial fibrillation ablation: how to manage?. Arrhythm Electrophysiol Rev.

[36] Bulava A., Hanis J., Dusek L. (2017). Clinical outcomes of radiofrequency catheter ablation of atrial fibrillation in octogenarians-10-year experience of a one high-volume center. J Geriatr Cardiol.

[37] Vermeersch G., Abugattas J.P., Varnavas V. (2021). Efficacy and safety of the second-generation cryoballoon ablation for the treatment of persistent atrial fibrillation in elderly patients. J Arrhythm.

[38] Müller J., Nentwich K., Berkovitz A. (2022). Efficacy and safety of high-power short duration atrial fibrillation ablation in elderly patients. J Cardiovasc Electrophysiol.

[39] Lerman T.T., Gadot C., Greenberg N. (2025). The Safety Profile of amiodarone among Older Adults (age ≥ 75 years): a pharmacovigilance Study from the FDA Data. Am J Med.

[40] Price S.D., Holman C.D.A.J., Sanfilippo F.M., Emery J.D. (2014). Impact of specific beers criteria medications on associations between drug exposure and unplanned hospitalisation in elderly patients taking high-risk drugs: a case-time-control study in Western Australia. Drugs Aging.

[41] Wijffels M.C., Kirchhof C.J., Dorland R., Allessie M.A. (1995). Atrial fibrillation begets atrial fibrillation. A study in awake chronically instrumented goats. Circulation.

[42] Goette A., Corradi D., Dobrev D. (2024). Atrial cardiomyopathy revisited-evolution of a concept: a clinical consensus statement of the European Heart Rhythm Association (EHRA) of the ESC, the Heart Rhythm Society (HRS), the Asian Pacific Heart Rhythm Society (APHRS), and the Latin American Heart Rhythm Society (LAHRS). Europace.

[43] Jastrzębski M., Kiełbasa G., Fijorek K. (2021). Comparison of six risk scores for the prediction of atrial fibrillation recurrence after cryoballoon-based ablation and development of a simplified method, the 0-1-2 PL score. J Arrhythm.

[44] La Rosa G., Morillo C.A., Quintanilla J.G. (2024). Practical approach for atrial cardiomyopathy characterization in patients with atrial fibrillation. Rev Esp Cardiol (Engl Ed).

[45] Calvo D., Filgueiras-Rama D., Jalife J. (2018). Mechanisms and drug development in atrial fibrillation. Pharmacol Rev.

[46] Popa M.A., Bourier F., Lengauer S. (2022). Safety profile and long-term efficacy of very high-power short-duration (60-70 W) catheter ablation for atrial fibrillation: results of a large comparative analysis. Europace.

[47] Boehmer A.A., Kaess B.M., Ruckes C. (2024). Pulmonary vein isolation or pace and ablate in elderly patients with persistent atrial fibrillation (ABLATE versus PACE)-rationale, methods, and design. Can J Cardiol.

[48] Calkins H., Hindricks G., Cappato R. (2017). 2017 HRS/EHRA/ECAS/APHRS/SOLAECE expert consensus statement on catheter and surgical ablation of atrial fibrillation: executive summary. J Arrhythm.

